# Protective effect of dexmedetomidine in cecal ligation perforation-induced acute lung injury through HMGB1/RAGE pathway regulation and pyroptosis activation

**DOI:** 10.1080/21655979.2021.2000723

**Published:** 2021-12-07

**Authors:** Huaqin Sun, Hongyi Hu, Xiaoping Xu, Mingsun Fang, Tao Tao, Zhehao Liang

**Affiliations:** a Department of Anesthesiology, The First Affiliated Hospital of Zhejiang Chinese Medical University, Hangzhou, Zhejiang Province, China; b Laboratory Animal Research Center, Academy of Chinese Medical Sciences, Laboratory Animal Research Center, Zhejiang Chinese Medical University, Hangzhou, Zhejiang Province, China; c Department of Ultrasound, The First Affiliated Hospital of Zhejiang Chinese Medical University, Hangzhou, Zhejiang Province, China

**Keywords:** Dexmedetomidine, acute lung injury, HMGB1; RAGE, NF-κB, pyroptosis

## Abstract

Dexmedetomidine (DEX) has been reported to attenuate cecal ligation perforation (CLP)-stimulated acute lung injury (ALI) by downregulating *HMGB1* and *RAGE*. This study aimed to further investigate the specific mechanisms of *RAGE* and its potential-related mechanisms of DEX on ALI models *in vitro* and *in vivo*. The *in vitro* and *in vivo* ALI models were established by lipopolysaccharide treatment in MLE-12 cells and CLP in mice, respectively. The effect of DEX on pathological alteration was investigated by HE staining. Thereafter, the myeloperoxidase (MPO) activity and inflammatory cytokine levels were respectively detected to assess the lung injury of mice using commercial kits. The expression levels of HMGB1, RAGE, NF-κB, and pyroptosis-related molecules were detected by RT-qPCR and Western blot. HE staining showed that lung injury, increased inflammatory cell infiltration, and lung permeability was found in the ALI mice, and DEX treatment significantly attenuated lung tissue damage induced by CLP. The MPO activity and inflammatory cytokines (TNF-α, IL-1β, and NLRP3) levels were also significantly reduced after DEX treatment compared with those in the ALI mice. Moreover, DEX activated the HMGB1/RAGE/NF-κB pathway and upregulated the pyroptosis-related proteins. However, the protective DEX effect was impaired by RAGE overexpression in ALI mice and MLE-12 cells. Additionally, DEX treatment significantly suppressed HMGB1 translocation from the nucleus region to the cytoplasm, and this effect was reversed by RAGE overexpression. These findings suggested that DEX may be a useful ALI treatment, and the protective effects on ALI mice may be through the inhibition of HMGB1/RAGE/NF-κB pathway and cell pyroptosis.

## Introduction

Acute lung injury (ALI) is a severe pulmonary illness caused by various etiologies (e.g., pathogen infection, sepsis, ischemia-reperfusion, and shock) [1]. Progressive ALI is also classified as moderate or severe acute respiratory distress syndrome (ARDS) [2]. Approximately 3 million patients in the USA experience ARDS per year, which accounts for up to 10% of intensive care unit admissions [3]. ALI mortality of ALIs is still high although treatments have greatly advanced. Available therapeutic options are limited beyond conservative respiratory support. However, mechanical ventilation with high oxygen concentrations may exacerbate the condition and further cause alveolar injury [4]. Thus, novel effective methods need to be currently developed for the ALI treatment.

ALI pathogenesis is involved in massive proinflammatory cytokine release and diffused alveolar epithelial cell injury [5]. Mechanically, the innate immune system defends against pathogen infection using a class of germline-encoded pattern recognition receptors, which could recognize multiple damage-associated molecular patterns (DAMPs) [6,7]. The recognition of DAMPs triggered inflammasome assembly in different cell types and then initiates caspase-dependent inflammatory processes [8,9]. High-mobility group protein 1 (HMGB1) is identified as a DAMP molecule-mediated inflammatory response in sepsis, cancer, and immune diseases. HMGB1 interacts with cell receptors, including receptor for advanced glycation end-product (RAGE), and toll-like receptor 2 (TLR2) and TLR4 and initiates a signaling cascade leading to nuclear factor-κB (NF-κB) pathway activation in ALI [10,11]. Moreover, diffuse cell injury is one of the most important pathological characteristics in ALI. Pyroptosis is a programmed cell death characterized by swelling and lysis, and excessive pyroptosis can lead to multiple organ dysfunction and septic shock [12]. Studies have recently demonstrated that increasing the level of cellular HMGB1 promotes pyroptosis and apoptosis to mediate organ injury in sepsis [13–15]. Therefore, targeting HMGB1/RAGE may be a potentially effective strategy for clinical therapy in sepsis. However, the effects of HMGB1/RAGE on cell pyroptosis in sepsis-induced ALI remain unclear.

Dexmedetomidine (DEX) is an agonist of α2-adrenergic receptors and is applied as an anxiolytic, sedative, and pain agent in clinical settings. Lipopolysaccharide (LPS), known as important inflammatory inducers, has been reported to treat with a murine lung epithelial (MLE) cell line MLE-12 to induce acute lung injury *in vitro* [16–18]. A growing number of studies showed that DEX has a potent anti-inflammatory effect that alleviates ALI through complex mechanisms in experimental models *in vivo* and *in vitro* [19–23]. For example, previous studies have shown that DEX could suppress the inflammatory response by inhibiting the production of proinflammatory cytokines (tumor necrosis factor (TNF)-α and interleukin (IL)-1β, IL-6), thus protecting against sepsis-induced ALI or acute kidney injury [24–26]. Moreover, DEX has also been reported to depress the translocation of HMGB1 from the nucleus to the cytoplasm and its exocrine secretion in LPS-activated macrophages, as well as the HMGB1 mRNA expression [27]. RAGE can interact with HMGB1, and a previous study found that the RAGE pathway participated in the actions of DEX on sepsis-stimulated ALI in rats [19]. However, the specific roles of RAGE in sepsis-induced ALI and its related mechanisms need to be further explored.

Therefore, the hypothesis that DEX may exhibit protective effects in the ALI model through the HMGB1/RAGE pathway and cell pyroptosis suppression is aimed to be verified in this study. *In vivo* ALI model was induced by cecal ligation perforation (CLP) in mice, and in vitro ALI model was established using LPS-treated MLE-12 cells. A novel RAGE-overexpressed vector was synthesized via recombinant DNA technology thereafter, and the ALI mice were treated with DEX or RAGE-overexpressed lentivirus to further investigate the specific regulatory mechanisms of RAGE/HMGB1 in sepsis-related ALI. This work will provide additional new insights into sepsis-related ALI management.

## Materials and methods

### Regents

DEX and LPS were purchased from Beyotime Biotech. (Nanjing, China). qPCR Kit (SYBR Premix Ex Taq) and RIPA lysis buffer were supplied by TaKaRa (Dalian, China). A reverse transcription kit was purchased from TransGen (Beijing, China). Enzyme-linked immunosorbent assay (ELISA) kits of tumor necrosis factor-alpha (TNF-α), interleukin IL-1β, and NLR family pyrin domain-containing 3 (NLRP3) were supplied by Elabscience Biotech. (Wuhan, Hubei, China). All antibodies, including caspase-11; Gasdermin D (GSDMD); HMGB1; RAGE; NF-κB; PYD; and CARD domain-containing ASC, NLRP3, caspase-1, GSDMD-N, and glyceraldehyde-3-phosphate dehydrogenase (GAPDH), were purchased from Abcam Inc. (Cambridge, UK).

### Animals

Sixty male C57BL/6 mice were purchased from Shanghai SLAC Laboratories Animal Co. Ltd. (Shanghai, China) and maintained at room temperature (25–27°C) for 1 week before surgical procedures. The mice were supplied with water and standard laboratory feeding condition. All procedures described in the current study were approved by the Ethical Committee for the Use of Laboratory Animals of The First Affiliated Hospital of Zhejiang Chinese Medical University, Hangzhou, China.

### Establishment of the ALI model

A CLP-induced ALI murine model was constructed as previously described [19,26]. First, the mice were anesthetized with 350 mg/kg chloral hydrate (i.p.), and the lower abdominal wall of the animal was shaved and cleaned with iodine solution. After a 2-cm incision at the midline abdominal cavity, the cecum was exposed and subsequently ligated using a 5–0 suture 1 cm distal to the cecal tip. The ligated cecum was punctured twice with a 21 G needle and gently pressed to overflow intestinal content afterward. The cecum was placed back into the abdominal cavity, and the incision was closed with sterile absorbable sutures. The anal temperature was maintained at 37 ± 0.5°C condition.

Thirty minutes after the surgical procedure, the mice were randomly divided into five groups as follows (*n* = 6): sham group, CLP group, CLP + DEX group (i.p. injected with 2.5, 5, and 10 μg/kg DEX). The DEX dosage was based on the previous study reported [19]. The animals were sacrificed to collect lung tissues and bronchoalveolar lavage fluid (BALF) after 24 h.

### HE staining, pathological scores, and MPO activity

Pulmonary samples were processed by formaldehyde fixation, ethanol dehydration, and embedding of paraffin following cutting into 4–6 μm thickness slices. The pathological change was observed under the microscope after standard HE staining protocol for lung tissue [28]. The lung injury grade was evaluated in differential groups following morphological changes, including hemorrhage, alveolar wall edema, and inflammatory cell infiltration.

The alveolar injury standard was also evaluated by a pathological scoring system based on previous references described [29,30]. Each sample could be scored between 0 and 4 (0–4 score represented normal, mild, medium, severe, and extremely severe injury). All the scores were evaluated by statistical analysis.

The lower lobes were cleaned and weighted on filter paper. The lung samples were reweighted as *dry* weight after storage at 80°C. Moreover, pulmonary edema severity was determined by calculating the wet/dry ratio of the lung lobes.

MPO activity in the lung tissues was also detected by using commercial test kits to explore the macrophages and neutrophils infiltration. The lung lobes were processed into 5% homogenates and incubated at 37°C water for 30 min. The enzymatic activity was exposed at 460 nm using a microplate reader.

### Inflammatory cytokines

The BALF mice samples were collected, and inflammatory cytokines were detected using ELISA kits (Elabscience Biotech, Wuhan, Science) following the manufacturer’s instructions, including TNF-α, IL-1β, and NLRP3. The detection ranges for TNF-α ELISA, IL-1β ELISA, and NLRP3 ELISA are 20–640, 3.75–120, and 25–800 pg/mL, respectively.

### RAGE overexpression mice model

The RAGE primer sequence was 5′-ACCGAGTCCGAGTCTACCAGATTC-3′ (forward) and 5′-CCACCTTCAGGCTCAACCAACAG-3′ (reverse). The RAGE cDNA was amplified via polymerase chain reaction (PCR). After digestion with the restriction enzyme, RAGE cDNA was inserted into the pEGFP-N1 vector (Invitrogen) at NheI and XhoI sites, generating a recombinant pEGFP-RAGE plasmid. The 293 T cells were cultured in Dulbecco’s modified eagle’s medium (DMEM) + 10% fetal bovine serum within 18 passages in these experiments. Once reaching 70%–90% confluence, 293 T cells were then transfected with pEGFP-RAGE to overexpress RAGE using HighGene transfection reagent (ABclonal Biotechnology, Wuhan, China). The filtered lentiviral particle was harvested by low-speed supernatant centrifugation and stored at −80°C after 48 h of incubation.

RAGE-overexpressed mice model was constructed by intravenous (i.v.) injection of the virus supernatants. The mice were divided into five groups (*n* = 6): sham, CLP, CLP + DEX, CLP + DEX + vector, and CLP + DEX + oeRAGE groups. The mice in the CLP + DEX + vector, and CLP + DEX + oeRAGE groups were i.v. injected with the vector virus supernatant (200 μL, MOI50) and RAGE-overexpressing lentivirus supernatant (200 μL, MOI50), respectively, 48 h before CLP modeling. The mice in all groups except for the sham group were thereafter used for CLP construction. The mice in the CLP + DEX, CLP + DEX + vector, and CLP + DEX + oeRAGE groups were all administrated with DEX (10 μg/kg) after 30 min of CLP. The lung tissues samples in the different groups were harvested after 24-h treatment. One part was used to evaluate the viral infection efficiency using quantitative real-time PCR (RT-qPCR), Western blot, and immunofluorescence assay (eGFP). However, another part was used to investigate the pathological changes, MPO activities, and protein levels in the different groups.

### RT-qPCR assay

The mRNA expression levels of *IL-1β, caspase-11, GSDMD, HMGB1, RAGE*, and *NF-κB* were detected by the qPCR assay. Total RNA was exacted from the mice lung tissues or MLE-12 cells using TRizol reagent and stored at −80°C. The extracted total RNA was then reversely converted into cDNA using the TransScript one-step RT-qPCR kit. Specific primers for murine genes were designed and produced in Genwiz Inc. (Suzhou, China) to amplify cDNA sequences. The primer sequences are listed in Table 1. RT-qPCR assays were conducted using an MX-3000P Real-time PCR device (Stratagene, La Jolla, CA, USA). Relative mRNA expression levels of target genes were evaluated by comparison with housekeeping gene *GAPDH*.

**Table 1.** The primer sequences in RT-PCR assay

Gene name	Primer sequences
IL-1β	Forward 5ʹ- −3ʹGAAATGCCACCTTTTGACAGTG
	Reverse 5ʹ- −3ʹTGGATGCTCTCATCAGGACAG
Caspase-11	Forward 5ʹ- −3ʹGGAATGTGCTGTCTGATGTCTG
	Reverse 5ʹ- −3ʹCTACGATGTGGTGGTGAAAGAG
Gasdermin D	Forward 5ʹ- −3ʹTGTCAACCTGTCAATCAAGGA
	Reverse 5ʹ- −3ʹAGCCAAAACACTCCGGTTC
HMGB1	Forward 5ʹ- −3ʹGCACAAGAAGAAGCACCCGG
	Reverse 5ʹ- −3ʹTTGGGTGCATTGGGGTCCTT
RAGE	Forward 5ʹ- −3ʹACCGAGTCCGAGTCTACCAGATTC
	Reverse 5ʹ- −3ʹCCACCTTCAGGCTCAACCAACAG
NF-κB	Forward 5ʹ- −3ʹAGCACAGATACCACCACCAAGACC
	Reverse 5ʹ- −3ʹGGGCACGATTGTCAAAGAT
GAPDH	Forward 5ʹ- −3ʹGAAGGTCGGTGTGAACGGATTTG
	Reverse 5ʹ- −3ʹCATGTAGACCATGTAGTTGAGGTCA

### Western blot

The explored protein expression levels of HMGB1 signaling proteins and pyroptosis-related proteins in lung tissues and murine lung cells were detected. Lung lobes in different groups were harvested and homogenized for protein extraction using lysis buffer. Protein concentrations were detected using a BCA assay kit (Thermo Fischer Scientific, Shanghai, China). After heating at 95°C for denaturation, the proteins were separated by 10% SDS-PAGE and processed by transmembrane, nonfat milk blocking and incubation of primary (caspase-11, GSDMD, ASC, caspase-1, GSDMD-N, HMGB1, RAGE, and p-NF-κB) and secondary antibodies. The blots were then detected by ECL methods and imaged using a gel imaging system.

### Cell culture and treatments

LPS is a membrane component of gram-negative bacteria and has been widely used to induce a lung injury model. The murine lung epithelial cell line MLE-12 (American Type Culture Collection, CRL-2110), a type II alveolar epithelial cell, was maintained at 37°C, 5% CO_2_ incubator and cultured in DMEM/F12 medium, containing 2% fetal bovine serum. Cells were cocultured with LPS (1 μg/mL) for 24 h to induce ALI cell model [16]. Cells were divided into the control (only LPS incubation), DEX (10 μM), DEX (10 μM) + empty vector, and DEX (10 μM) + RAGE overexpression groups after reaching approximately 80% confluence. The cells were changed with a fresh culture medium and added screening antibiotics (G418 or puromycin) after lentivirus infection for 18 h. The cells were stimulated for 24 h before RNA and protein harvesting. The culture supernatant was collected for cytokine detection of TNF-α, IL-1β, and NLRP3.

### Immunofluorescence assay

MLE-12 cells were fixed with 4% formaldehyde and processed using 1% Triton X-100 for cell penetration. The samples were incubated with primary and secondary antibodies after blocking in 1% bovine serum albumin solution for 1 h. Cell nuclei areas were stained using DAPI. The coverslips were ready and fluorescence images were taken using a PerkinElmer microscope (Waltham, MA, USA).

### Statistical analysis

Data were shown as mean ± standard deviation (SD). Statistical analysis was conducted using GraphPad 7.0 software (GraphPad Software Inc., San Diego, CA, USA). The homogeneity of variance test for all data was performed and the *F* and *P* values were calculated before the analyses. A one-way analysis of variance (ANOVA) followed by Tukey’s test was applied for multiple comparisons if *P* value > 0.05; Brown–Forsythe and Welch ANOVA tests were used if *P* value ≤ 0.05. *P* < 0.05 was considered statistically significant.

## Results

### DEX-alleviated lung injury in the CLP-induced mice model

**Figure 1.**
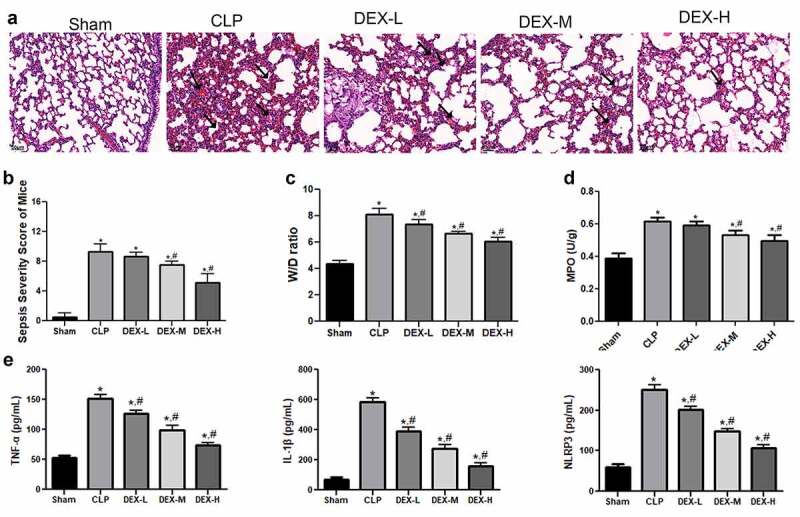
**DEX protected against lung injury in CLP-induced mice model.** (a) Pathological changes of the lung samples in CLP-induced mice. Representative HE staining of lung tissue slices from sham, CLP, and different DEX doses groups. *DEX-L* low dose of DEX (2.5 μg/kg), *DEX-M* medium dose of DEX (5 μg/kg), and *DEX-H* high dose of DEX (10 μg/kg). *Scale bars* 50 µm. (b) Pathological score of the lung sample in each group. (c) The W/D weight ratio of the lung in each group. (d) MPO activity of the lung samples in mice. (e) Inflammatory cytokines level of TNF-α, IL-1β, and NLRP3 in BALF sample. The mice were i.p. injected with 2.5, 5, and 10 μg/kg of DEX 30 min after CLP procedure. Three animals in each group were sacrificed to collect lung and BALF after 24 h. *N* = 6. Data were expressed as mean ± SD. *P < 0.05, *vs*. sham; ^#^*P* < 0.05, *vs*. CLP mice

Different DEX doses were used to treat CLP-induced mice, and HE staining was used to observe the lung injury of mice in the different groups. Mice in the sham group exhibited no lung injury, while the CLP-induced mice exhibited notable histological changes in lung architecture, extensive pulmonary hemorrhage, and increased inflammatory cell infiltration (Figure 1a). The severity of lung injury induced by CLP was significantly attenuated in a dose-dependent manner after different doses of DEX treatment (Figure 1a).

The sepsis severity score was calculated in each group thereafter. Figure 1b shows that the pathological score of the ALI mice significantly increased compared with that in the sham group (*P* < 0.05). The pathological score was significantly decreased in a DEX dose-dependent manner after DEX treatment (Figure 1b). The findings of the current study indicated that DEX treatment could reduce lung pathological injury in CLP-induced ALI mice.

The wet/dry (W/D) weight ratio of lung tissue was also detected to estimate pulmonary edema severity. The W/D ratio of the CLP-induced mice significantly increased compared with the sham group (Figure 1c, *p* < 0.05). Moreover, DEX treatment significantly decreased the W/D weight ratio in a dose-dependent manner.

MPO activity, a biochemical marker of neutrophil infiltration, was determined in the lung tissue samples. Figure 1d shows that the CLP-induced mice exhibited significantly higher MPO activity than the mice in the sham group (*P* < 0.05). The MPO activity was gradually reduced and the difference reached a statistically significant level in the medium- and high-dose (5 and 10 μg/kg DEX) groups compared with that in the CLP-induced group after DEX treatment (*P* < 0.05).

In addition, inflammatory TNF-α, IL-1β, and NLRP3 cytokines are responsible for immune response in ALI models. The CLP-induced ALI mice exhibited increased levels of these cytokines in the BALF samples (*P* < 0.05). Inflammatory cytokine levels were notably reduced after treatment with different DEX levels (Figure 1e, *p* < 0.05). These changes indicated that DEX could protect against CLP-induced ALI by decreasing the release of TNF-α, IL-1β, and NLRP3.

### Viral infection efficiency

**Figure 2.**
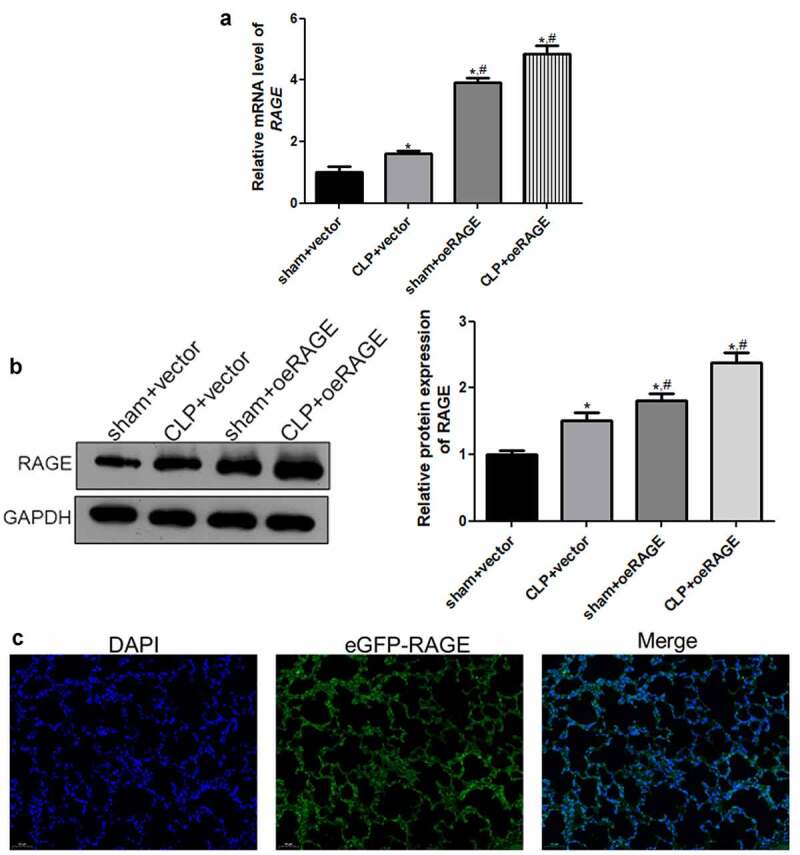
**The viral infection efficiency after RAGE overexpression mice construction.** (a) The relative mRNA expression of *RAGE* after infection using RT-qPCR. (b) The relative protein RAGE expression after infection by Western blot. (c) The eGFP-oeRAGE expression in the lung tissues of the RAGE overexpression mice. *N* = 6. Data were expressed as mean ± SD. *P < 0.05 *vs*. sham + vector; ^#^*P* < 0.05 *vs*. CLP + vector

A RAGE-overexpressed mice model was established by injection of RAGE-overexpressing lentivirus and infection efficiency was assessed to further investigate the roles of RAGE in sepsis-induced ALI. RT-qPCR and Western blot showed that the relative RAGE expression was significantly increased after RAGE-overexpressing lentivirus infection compared with the vector groups (*P* < 0.05, Figure 2a, b). Moreover, the immunofluorescence results of eGFP displayed that the green fluorescence of eGFP-RAGE existed in the lung tissues of the CLP + DEX +oeRAGE group (Figure 2c), which indicated that RAGE was significantly expressed in the lung tissues after RAGE-overexpressing lentivirus infection. All the results indicated that the RAGE overexpression mice model was successfully established and could be used for further experiments.

### RAGE overexpression impaired protective effect of DEX

**Figure 3.**
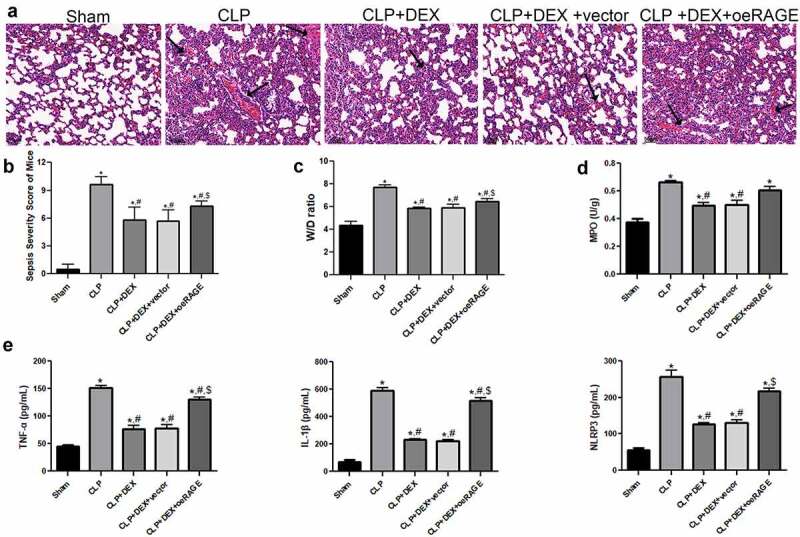
**Protective effect of DEX on the pathological manifestation of lung tissue could be impaired by injection of RAGE lentiviral**. (a) Representative HE staining of lung tissue slices from sham, CLP, CLP + DEX (10 μg/kg), and CLP +DEX + RAGE overexpression (oeRAGE) group. *Scale bars* = 50 µm. RAGE-overexpressed mice model was constructed by i.v injection of RAGE-overexpressed lentivirus supernatants. (b) Pathological score of lung samples in each group. (c) The W/D weight ratio of the lung in each group. (d) MPO activity of the lung sample in mice. (e) Inflammatory cytokine release of TNF-α, IL-1β, and NLRP3 in BALF sample of mice. RAGE-overexpressed mice model was constructed by i.v injection of RAGE-overexpressed lentivirus supernatants. The mice were treated with DEX (10 μg/kg) 30 min after the CLP procedure. Three animals in each group were sacrificed to collect BALF samples after 24 h. = 6. Data were expressed as mean ± SD. *P < 0.05 *vs*. sham; ^#^*P* < 0.05 *vs*. CLP mice; ^$^*P* < 0.05, *vs*. CLP + DEX

The pathological changes in the RAGE-overexpressed mice were evaluated by HE staining (Figure 3a). The CLP-induced mice notably exhibited alveolar damage and inflammatory cell infiltration in the lung tissues. The DEX protective effects were partly impaired by injection of RAGE-overexpressed lentiviral and resulted in obvious alveolar hemorrhage and significant neutrophil infiltration (Figure 3a). The pathological score in the different groups was then calculated, and the results were consistent with the morphological changes determined by HE staining (Figure 3b). Moreover, the major manifestations of RAGE-overexpressed mice were explored. It showed that RAGE overexpression partly reversed the reduction of W/D weight ratio and MPO activity induced by DEX treatment (*P* < 0.05, Figure 3c, d). RAGE overexpression also significantly increased the releases of inflammatory cytokines (TNF-α, IL-1β, and NLRP3) in BALF compared with the DEX (10 μg/kg) and DEX+ vector groups in the ELISA assays (*P* < 0.05, Figure 3e). Taken together, these results indicated that the protective role of DEX in ALI could be impaired by RAGE overexpression.

### Alterations of the HMGB1/RAGE and pyroptosis pathway in RAGE-overexpressed mice

**Figure 4.**
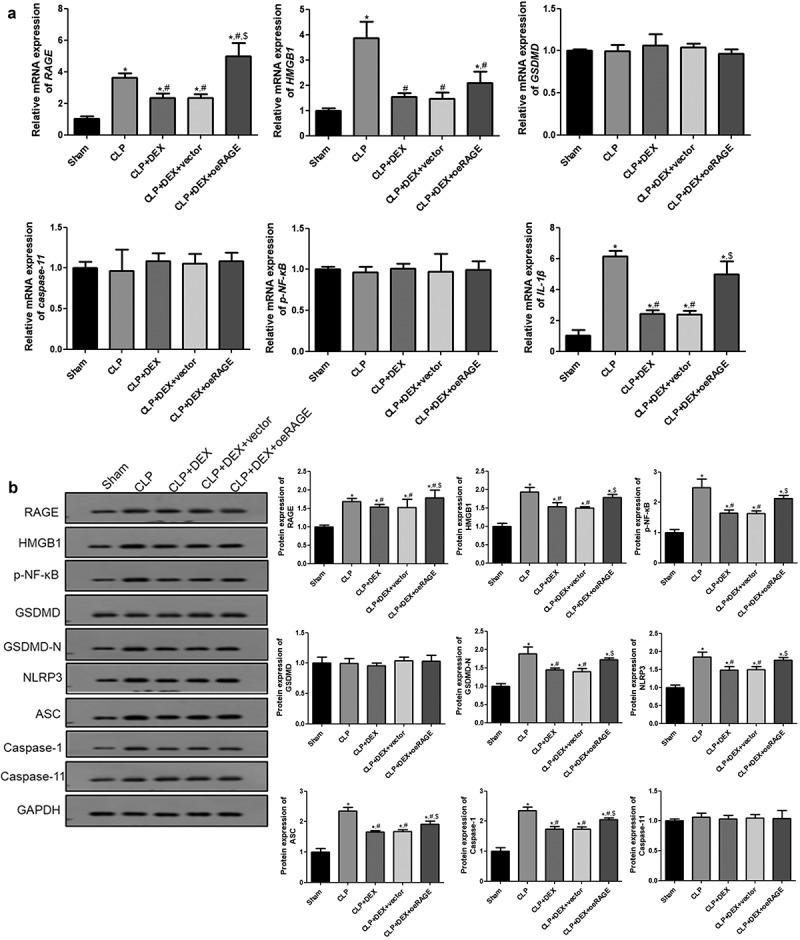
**RT-qPCR** (a) **and Western blot** (b) **analyses of HMGB1/RAGE pathway and pyroptosis-related proteins in mice**. The expression of mRNAs and proteins was normalized with a housekeeping gene *GAPDH*. ˆ = 6. Data were expressed as mean ± SD. *P < 0.05, *vs*. sham; ^#^ *P* < 0.05, *vs*. CLP mice; ^$^*P* < 0.05, *vs*. CLP + DEX

The expression levels of RAGE and pyroptosis-related factors in each group were examined by RT-qPCR and Western blot to further explore the molecular mechanisms of DEX in ALI. Figure 4a shows that the mRNA expressions of *RAGE, HMGB1*, and *IL-1β* were significantly increased in the CLP group but significantly decreased after DEX treatment (*P* < 0.05). Moreover, the RAGE overexpression further increased the mRNA expression levels of these genes, while no effects of empty vectors were detected (*P* < 0.05). However, the mRNA expression levels of caspase-11, GSDMD, and p-NF-κB were found to not significantly change among all groups (*P* > 0.05; Figure 4a).

Additionally, the protein levels of RAGE, HMGB1, p-NF-κB, and pyroptosis-related factors, including GSDMD, GSDMD-N, NLRP3, ASC, caspase-1, and caspase-11, were further detected by Western blot (Figure 4b). The protein levels of RAGE, HMGB1, and p-NF-κB were significantly upregulated in the CLP group compared with those in the sham group (*P* < 0.05) but significantly decreased in DEX treatment groups (*P* < 0.05). RAGE overexpression partly reversed the decrease of HMGB1 and p-NF-κB (*P* < 0.05). For pyroptosis-related factors, GSDMD-N, NLRP3, ASC, and caspase-1 were significantly upregulated in the CLP group compared with those in the sham group (*P* < 0.05) but significantly downregulated after DEX treatment (*P* < 0.05). RAGE overexpression could partly counteract the effect of DEX treatment (*P* < 0.05). The GSDMD and caspase-11 protein levels were not significantly changed among all groups (*P >* 0.05).

### DEX inhibited the release of inflammatory cytokines in LPS-treated MLE-12 cell

**Figure 5.**
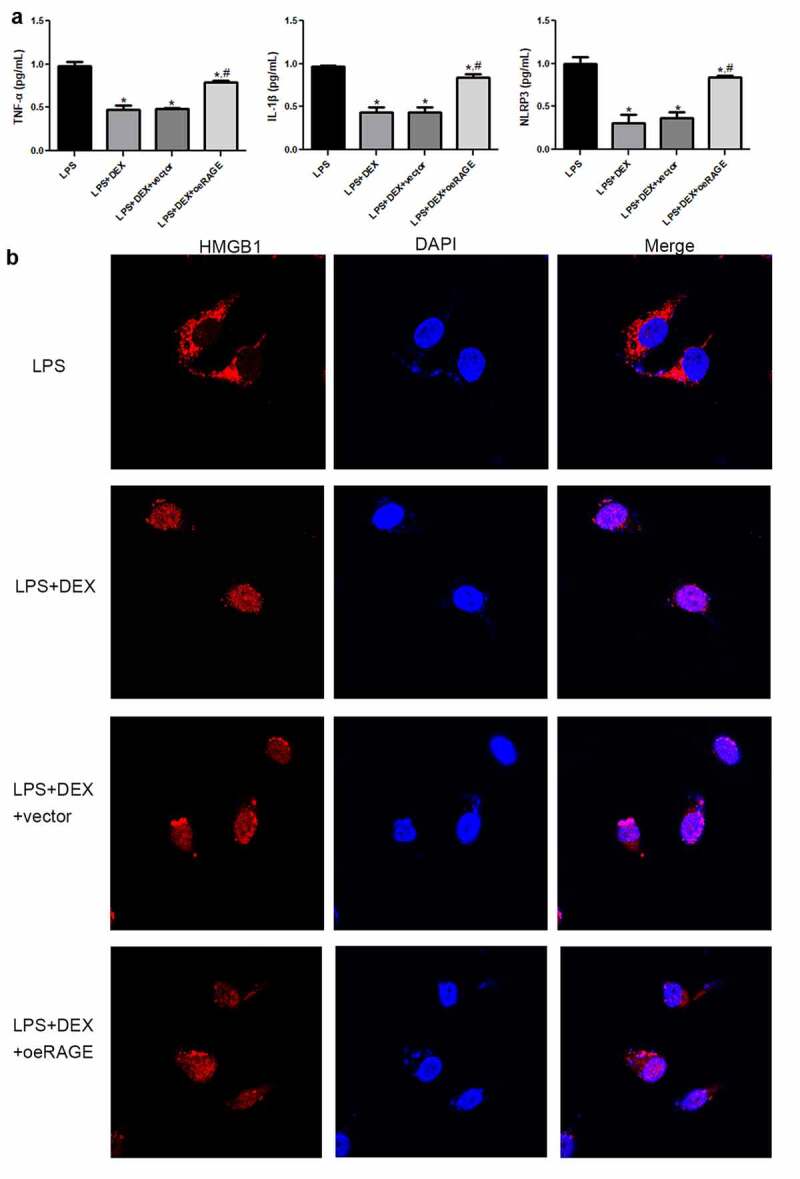
**Inflammatory cytokine release in LPS-exposed MLE-12 cell** (a) **and** immunofluorescence analysis of HMGB1 location in LPS-stimulated MLE-12 cells (b). After 24 h of LPS (1 μg/mL) exposure, the MLE-12 cells were treated with DEX (10 μM) or RAGE-overexpressed lentivirus for 18 h. Subsequently, cell supernatant was collected, and inflammatory cytokines were determined by ELISA. *N* = 6. Data were expressed as mean ± SD. *P < 0.05, *vs*. LPS; ^#^*P* < 0.05, *vs*. LPS + DEX

In addition, an *in vitro* ALI model was established using LPS-treated MLE-12 cells, and the molecular mechanism of DEX on ALI was then investigated. Figure 5a shows that the inflammatory cytokines, including TNF-α, IL-1β, and NLRP3, were significantly decreased in the DEX-treated group compared with those in the LPS group (*P* < 0.05). However, the inhibitory effects on cytokine release were abrogated by RAGE-overexpressed lentivirus coincubation (*P* < 0.05).

### DEX affected the HMGB1 transportation to the nucleus in LPS-treated MLE-12 cell

An immunofluorescence staining assay was applied to determine the HMGB1 location in the LPS-stimulated MLE-12 cells (Figure 5b). The results showed that HMGB1 was distributed in the cytoplasm of LPS-treated MLE-12 cells. HMGB1 was mostly expressed in the nucleus after DEX treatment. However, HMGB1 transportation into the nucleus was inhibited by RAGE-overexpressed lentivirus.

### DEX decreased HMGB1/RAGE levels and pyroptosis-related proteins in the LPS-treated MLE-12 cell

**Figure 6.**
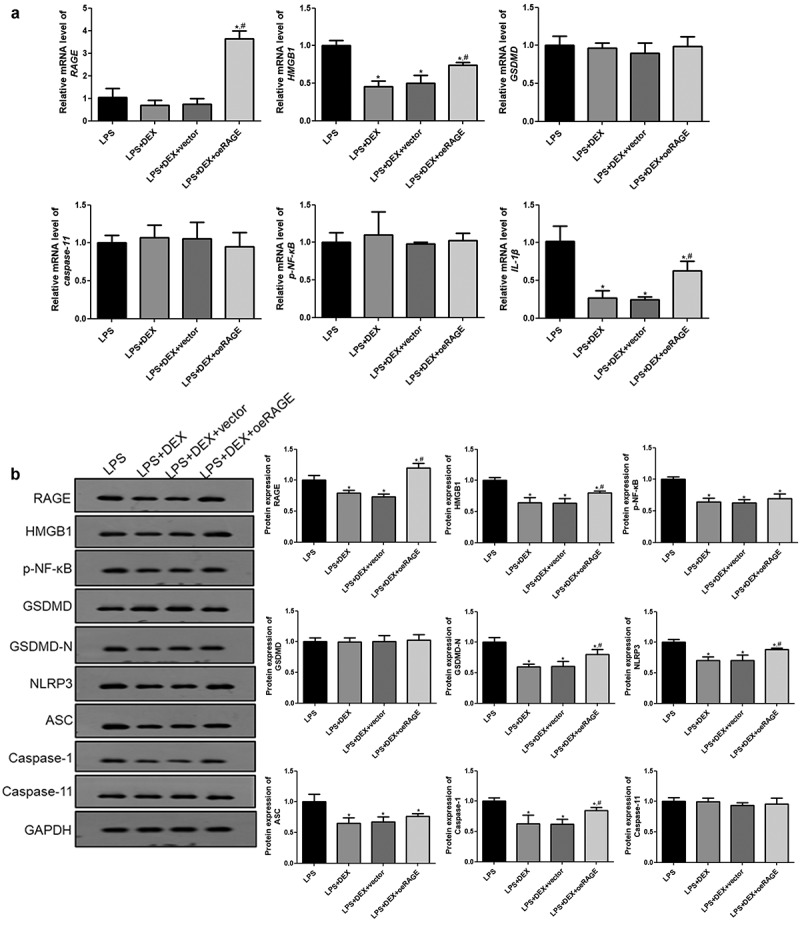
**RT-qPCR** (a) **and Western blot** (b) **analyses of HMGB1/RAGE pathway and pyroptosis-related proteins in LPS-treated MLE-12 cell**. *N* = 6. Data were expressed as mean ± SD. ^#^*P* < 0.05, *vs*. LPS; ^$^*P* < 0.05, *vs*. LPS + DEX

Finally, the mRNA expression levels of the genes involved in the HMGB1/RAGE pathway and cell pyroptosis in the LPS-induced MLE-12 cells were examined using RT-qPCR and Western blot. Figure 6a shows that DEX significantly downregulated the expressions of *HMGB1* and *IL-1β* (*P* < 0.05). RAGE overexpression abolished some of the DEX inhibition effects on the expressions of HMGB1 and IL-1β (*P* < 0.05), while the empty vector was not (*P* > 0.05). However, no significant differences in the expression levels of *GSDMD, caspase-11*, and *NF-κB* among all groups were noted.

Furthermore, the protein expression levels of related proteins were detected. The results showed that DEX incubation significantly decreased the protein levels of caspase-1, HMGB1, RAGE, p-NF-κB, NLRP3, ASC, caspase-1, and GSDMD-N compared with the LPS group (Figure 6b, *p* < 0.05), whereas coincubation of DEX- and RAGE-overexpressed lentivirus notably upregulated these protein levels compared with the RAGE empty vector group (*P* < 0.05).

## Discussion

ALI and its severe form, ARDS, are common and clinically serious complications, affecting people’s health and life [31]. DEX has been reported to have protective effects on sepsis-stimulated ALI [19]. However, the specific related potential mechanisms have not been fully elucidated. The protective effects of DEX on ALI were validated in the CLP-stimulated mice model and LPS-treated MLE-12 cells in this study, and RAGE overexpression lentivirus was established. DEX treatment was found to significantly alleviate the lung histopathological damage, W/D weight ratio, MPO activity, and decreased inflammatory cytokine level induced by CLP. Moreover, DEX downregulated the expressions of the HMGB1/RAGE pathway-related genes/proteins and pyroptosis-related genes/proteins both in the CLP-stimulated ALI and LPS-induced MLE-12 cell models, whereas RAGE overexpression could impair the protective effects of DEX on ALI in the *in vivo* and *in vitr*o models.

A growing number of studies have shown that the release of inflammatory cytokines is an essential characteristic of lung parenchyma injury [26], and various inflammatory mediators, including IL-1s, TNF-α, and IL-6, are increased both in the BALF and circulating plasma of ARDS patients [32,33]. The elevated levels of inflammatory cytokines can initiate the inflammatory cascade and then induce neutrophil infiltration and promote lung damage [34]. A previous study by Kawasaki et al [24]. showed that DEX had inhibitory effects on inflammatory mediator production in human whole blood after LPS stimulation via the α2-adrenergic receptors and NF-κB inhibition. Another study has also indicated that DEX could reduce the mortality rate and inhibit the proinflammatory cytokine responses during polymicrobial sepsis in mice [26]. The cytokine levels of IL-1β and TNF-α in BALF samples in the current study significantly decreased in the DEX group compared with the control group. Moreover, MPO activity served as a marker of neutrophil infiltration that represented lung tissue damage in LPS-induced ALI [35]. Herein, DEX treatment notably reduced the major manifestations of lung samples. Duan et al [36]. demonstrated that narciclasine could protect against lung damage via the inhibitory effects of excessive inflammation (suppression of the secretion of IL-1β, TNF-α, and IL-6), oxidative stress, and cell apoptosis. Taken together, DEX can be speculated to possibly mitigate ALI via decreasing the production of proinflammatory cytokines (IL-1β and TNF-α).

NLRP3 forms an intracellular inflammasome complex, consisting of NLRP3, the adaptor protein, and caspase-1 [37]. NLRP3 inflammasome is an essential component in the innate immune system and responds to a wide range of pathogen infections and stress stimuli. The activation of the NLRP3 inflammasome typically requires two signal models [38]. The primary signal is induced by pathogen infection or cytokines, leading to NF-κB signaling activation; the second signal is triggered by wide cellular danger stimuli involved in multiple signaling events (e.g., K+ efflux, Ca^2+^ influx, mitochondrial destruction, and lysosomal leakage) [39,40]. Caspase-1 subsequently cleaves substrates into mature molecules, inducing the inflammation process, when NLRP3 inflammasome is activated [41]. Moreover, the substantial data suggested that NLRP3 inflammasome-mediated inflammatory responses are crucial for ALI development [42,43]. Additionally, the NLRP3 and p-NF-κB protein levels decreased in the DEX treatment group. Combined with the results of the current study, DEX may regulate the inflammatory response by inhibiting the NLRP3 inflammasome and NF-κB signaling pathway, thus alleviating ALI.

HMGB1 is a multifunction protein participating in various cellular processes (e.g., cell proliferation, inflammation, and oxidative stress). As a late inflammatory mediator, HMGB1 induces downstream signal cascade via interaction with cell receptors (RAGE, TLR2, and TLR4), leading to activation of ROS, MyD88, NF-κB, and PI3K pathways [44]. Inflammatory cytokines release further promotes the expression of HMGB1 that amplified the inflammatory response in ALI [45,46]. The current study focused on the HMGB1/RAGE/NF-κB pathway. The results showed that DEX treatment could significantly suppress the expression levels of HMGB1, RAGE, and p-NF-κB, which was consistent with previous studies [47,48]. The specific DEX mechanisms were further investigated by generation RAGE-overexpressed mice. The protective effects of DEX on ALI were notably alleviated by RAGE overexpression lentivirus injection. Previous studies showed that RAGE knockout or inhibitors application could suppress the cascade effects in inflammation disease and cancer [49,50]. Considering this, DEX was further confirmed to protect against ALI through suppression of the HMGB1/RAGE/NF-κB pathway.

Extracellular HMGB1 could be released from necrosis cells or secreted by activated immune cells. HMGB1 release is initiated with translocation from the nuclear region to the cytosol [51]. The location determined its biological function and HMGB1 is regulated by post-translational modification [52]. Moreover, HMGB1 consists of 215 amino acid residues, including two nuclear localization sites (NLSs) that regulated nuclear localization [53]. Under physiological conditions, HMGB1 is anchored inside the nucleus through the binding of NLS sites and the nuclear cargo [52]. Acetylation, phosphorylation, or methylation at NLSs results in dissociation of HMGB1 with anchored proteins, which is critical for its translocation from the nucleus to the cytosol in immune cells and other cell types. For example, hyperacetylated lysine in NLS sites promotes the nucleus to cytoplasm translocation of HMGB1 in LPS-stimulated monocytes [51]. Histone deacetylase activity affects the HMGB1 release from hepatocytes during the ischemia-reperfusion injury process [54]. Lu et al. showed that HMGB1 cytoplasmic translocation is regulated by the activation of the JAK/STAT1 signaling pathways [55]. Another group showed that IRF1 interacted with nuclear histone acetyltransferase enzyme p300 regulated HMGB1 acetylation of hepatocytes in murine ischemia-reperfusion damage [56]. The current study revealed that DEX treatment could significantly reduce HMGB1 location in the cytoplasm of MLE-12 cells after LPS stimulation.

In addition, pyroptosis and related inflammatory caspases were reported as major factors in ALI development [57]. The activation model of pyroptosis mainly consists of caspase-1-mediated canonical and caspase-11 or −4/5 mediated noncanonical pathways [58]. GSDMD is a specific substrate of these inflammatory caspases (caspase-1, caspase 11 in murine, and caspase 4/5 in humans), which could be cleaved into pore-forming peptides GSDMD-N, resulting in membrane rupture and pyroptosis-induced cell death [59,60]. Deng et al. recently discovered that hepatocyte-released HMGB1 was required for caspase-11-dependent pyroptosis in endotoxemia [61]. In addition, Ji et al. reported that DEX can decrease HMGB1-induced cell pyroptosis in trauma-derived inflammation [62]. In sepsis-induced brain injury, DEX protected astrocytes against pyroptosis, and the neuroprotective effect was abolished by α2-adrenoceptor antagonist [63]. Additionally, the current study also demonstrated that DEX inhibited the expression of caspase-1 and GSDMD-N in the LPS-stimulated MLE-12 cells. Therefore, the protective effects of DEX may be associated with the inhibition of caspase-1-mediated cell pyroptosis.

However, this study has some limitations. First, the beneficial roles of DEX against CLP-stimulated ALI should be further determined by the α2-AR antagonist or verified in the transgenic mice. Second, the correlations between HMGB1 and RAGE, as well as the mechanistic links between HMGB1/RAGE signaling and protective effects require more investigation not only in the alveolar epithelial cells but also in the immunity cells. The downstream mechanisms should also be completely concerned with more signaling pathways because ALI is a complex inflammatory process and the current study only focused on HMGB1/RAGE.

## Conclusion

In conclusion, this study confirmed that DEX had a protective effect on ALI and may affect the inflammatory response through the inhibition of the HMGB1/RAGE/NF-κB pathway and cell pyroptosis. The current study provides an experimental basis for further understanding the mechanism of DEX on ALI.

## Data Availability

All data generated or analyzed during this study are available from the corresponding author upon reasonable request.

## Highlights

- Dexmedetomidine possessed protective effects on acute lung injury *in vitro* and *in vivo*.
- Dexmedetomidine may alleviate ALI through regulation of the inflammatory response and cell pyroptosis.
- Dexmedetomidine may protect against ALI through the HMGB1/RAGE/NF-κB pathway suppression.
- Dexmedetomidine promoted HMGB1 translocation from the cytoplasm to the nucleus in LPS-activated MLE-12 cells.
